# Epidemiology and diagnosis of gout in sub-saharan Africa: a scoping review

**DOI:** 10.1186/s41927-024-00391-w

**Published:** 2024-05-23

**Authors:** Ayouba Tinni Ismael, Kabore Fulgence, Bayala Yannick Laurent Tchenadoyo, Yameogo Wendyam Nadège, Zabsonre/Tiendrebeogo Wendlassida Stéphanie Joelle, Ouedraogo Aboubakar, Zongo Yamyelle Enselme, Traore Awa, Bonkoungou Marcellin, Ouedraogo Dieu-Donné

**Affiliations:** 1 Rheumatology Department, Bogodogo University Hospital, Ouagadougou, Burkina Faso; 2 Joseph KI-ZERBO University, Ouagadougou, Burkina Faso

**Keywords:** Gout, Uric acid, Epidemiology, Diagnosis, Sub-saharan Africa

## Abstract

**Background:**

The episodic nature of gout and diagnostic uncertainty in the absence of microcrystal evidence make it particularly difficult to estimate the frequency of gout. Our aim was to review the literature on the epidemiological and diagnostic aspects of gout in sub-Saharan Africa.

**Methods:**

This literature review was conducted using the MEDLINE database (via PUBMED), Google Scholar, and conference abstracts. The selection process was based on reading the titles first, then the abstracts, and then the full texts once the articles had been selected. Studies were included in this review if they presented original findings on the epidemiological and/or diagnostic aspects of gout in sub-Saharan Africa. Two groups of two investigators independently reviewed the studies. The results were analysed descriptively.

**Results:**

The literature search identified 131 articles and 22 conference abstracts. Nineteen articles were included in our review. Twelve studies were retrospective, five were cross-sectional, one was prospective, and one was both retrospective and cross-sectional. The duration of the studies ranged from 1 to 15 years, and the sample size ranged from 15 to 511 patients, for a total of 2557 patients. Gout was quite common, with a maximum frequency of 11.87%. Fourteen articles diagnosed gout via criteria, including 9 studies totaling 1174 patients via the 1977 ACR criteria. Gout tophi were reported in 15 articles involving 464 patients. Of these studies, seven looked for monosodium urate crystals in 317 (43.85%) of 723 patients. Among the 317 patients, monosodium urate crystals were detected in 263 (82.97%) patients. Eleven studies reported mean uricemia values ranging from 452.09 µmol/L to 642.44 µmol/L, with a mean of 510.63 µmol/L.

**Conclusions:**

This review revealed that all the studies conducted in sub-Saharan Africa were intrahospital studies, and the majority were retrospective. Consequently, there is a clear need for population-based studies.

**Supplementary Information:**

The online version contains supplementary material available at 10.1186/s41927-024-00391-w.

## Background

Gout, one of the most common forms of inflammatory arthritis in middle-aged men, is caused by the deposition of monosodium urate (MSU) crystals in joints and soft tissues [1–5]. It has been known since ancient times, and its fame throughout history is inextricably linked to the great people who suffered from it [5]. Research in recent years has shown that sub-Saharan Africa is not spared from gout. Most of this work has been based on hospital consultations in urban areas. Although the results cannot be generalized to the whole population, these studies show the importance of gout in African rheumatic pathology [5]. In addition, the episodic nature of gout and diagnostic uncertainty in the absence of microcrystal evidence make it particularly difficult to estimate the frequency of gout [6]. In Ivory Coast, the frequency of gout was estimated at 2.55% out of 4157 rheumatologic diseases registered during the study period [7], and similarly, in Burkina Faso, it was estimated at 1.8 out of 23,550 patients during the study period [8]. The diagnostic difficulties in sub-Saharan Africa and the difference in frequencies reported in the literature guided this literature review, the aim of which was to summarize the studies that have addressed the epidemiological and diagnostic aspects of gout in sub-Saharan Africa.

## Methods

Due to the diversity of study designs on gout in sub-Saharan Africa and the lack of data synthesis, we decided to conduct a scoping review on the epidemiological and diagnostic aspects of gout in sub-Saharan Africa. The variables analysed were frequency and/or prevalence of gout, study design, diagnostic criteria, presence of tophi, and search for crystals.

### Study design

This systematic literature review was conducted using the MEDLINE database (via PubMed), Google Scholar, and conference abstracts for publications on the epidemiological and diagnostic aspects of gout in sub-Saharan Africa from 1966 to March 2023 in English or French. This search was supplemented by a manual search of the bibliographic references of all potentially eligible full-text articles.

Publications were identified using the keywords below in PubMed:

((gout[MeSH Terms] OR acute gout[MeSH Terms] OR microcrystalline arthritis[MeSH Terms] OR hyperuricemia[MeSH Terms] OR podagra[MeSH Terms] OR colchicine[MeSH Terms] OR allopurinol[MeSH Terms]) AND (Sub-Saharan Africa[MeSH Terms] OR Black Africa[MeSH Terms] (west Africa[MeSH Terms] OR Central Africa[MeSH Terms] OR East Africa[MeSH Terms] OR South Africa[MeSH Terms] OR Southern Africa[MeSH Terms]))

### Study selection

The selection process was based on reading the titles first, then the abstracts, and then, once the articles were selected, the full texts. The review was conducted with reference to the PRISMA-ScR statement (Preferred Reporting Items for Systematic Review and Meta-Analysis Protocols Extension for Scoping Reviews) [9]. The studies included in this review were published in peer-reviewed journals and were limited to observational studies using retrospective, cross-sectional, and/or longitudinal data. Conference abstracts published in peer-reviewed journals and not published as original articles were also considered for inclusion. The quality of the included studies was not assessed. Two groups of two reviewers (IAT, KF, and YLTB, WNY) independently evaluated the title, abstract, and full text of the articles. In the event of disagreement, a consensus was reached through discussion or the intervention of another person (D-DO).

### Inclusion and exclusion criteria

Studies on gout were included if they presented data on patients in whom the diagnosis of gout was retained on the basis of clinical and paraclinical arguments. These included studies that presented original results relating to one of two aspects of gout in sub-Saharan Africa: epidemiological (frequency and/or prevalence) and/or diagnostic (clinical and/or paraclinical). Studies presenting results concerning pharmacological, non-pharmacological, and surgical therapeutic methods for gout were excluded. Similarly, case reports, case series with fewer than five cases, and review articles were excluded.

### Information collected

The following data were extracted: year of publication, author’s name, study design, country in which the study was conducted, and patient characteristics: sample size, sex, mean age, disease duration, and diagnostic methods. The data extraction process was conducted using a preestablished form in an Excel spreadsheet. The data were extracted from one individual (IAT) and subsequently verified by a second individual (YLTB).

### Data analysis

The results concerning the epidemiological and diagnostic aspects of gout were summarized descriptively and are presented in a table. Where necessary, frequencies and means were used to summarize categorical and continuous variables. The data analysis was performed using R software version 4.2.3.

### Bias

The frequency of gout in this scoping review may be biased due to the retrospective nature of 12 of the 19 articles included in the data analysis. Indeed, the risks of bias in a retrospective study may include attrition bias due to missing data at follow-up, selection bias due to restrictive inclusion criteria, and measurement bias due to the accuracy of retrospectively collected data.

## Results

### Study selection

**Fig. 1:**
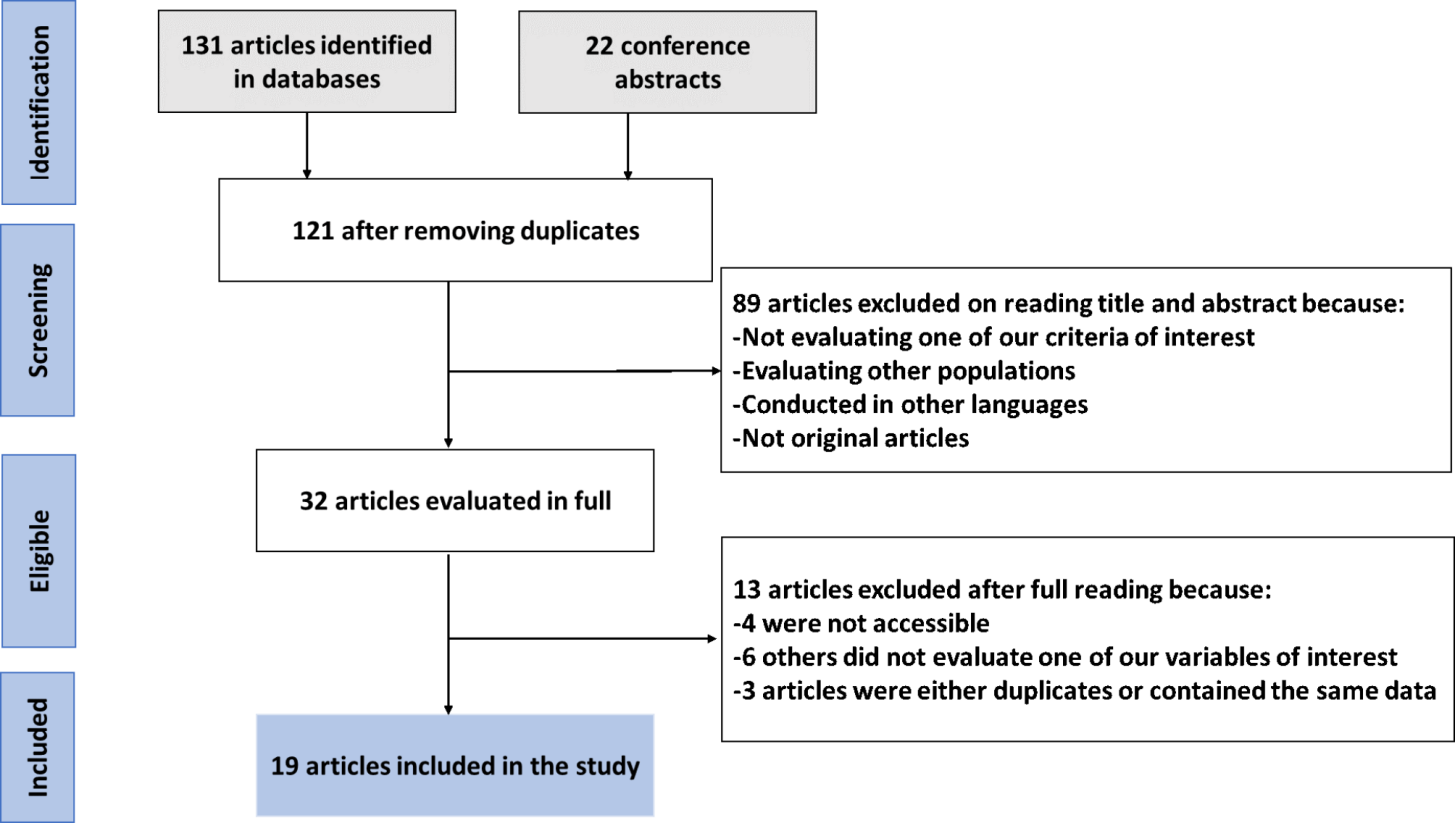
Flow chart

The literature search identified 131 articles and 22 conference abstracts. After removing 32 duplicates, 89 articles were excluded after reading the title and abstract (because they did not meet one of our criteria of interest, assessed other populations, were published in other languages, or were not original articles). Thirteen articles were also excluded after full reading because four of them were not accessible (a request had been sent to the authors), six others did not assess one of our variables of interest, and three articles were either duplicates or used the same data. Finally, 19 studies met the criteria [7, 10–27] and were included in the review (including seven conference abstracts published in peer-reviewed journals [10–16]). Figure 1 shows a flow chart of the literature search.

### Characteristics of the selected studies

Of the 19 studies included in our literature review, 12 were retrospective studies, five were cross-sectional studies, one was a prospective study, and one was a retrospective and cross-sectional study. Of the 19 studies, 11 were conducted in West Africa, seven of which were retrospective studies. Five of the studies were carried out in Central Africa, three of which were cross-sectional and two were retrospective. Three studies were conducted in southern Africa, all of which were retrospective. The duration of the studies ranged from one to 15 years. The number of gout patients included in the study ranged from 15 to 511, resulting in a total of 2,557 patients with a diagnosis of gout. The greatest number of gout patients was reported in Cameroon (511 gout patients) and Burkina Faso (425 gout patients) [10, 11]. Table 1 presents the various characteristics of the studies included in this scoping review.

**Table 1:** Summary of the characteristics of the different articles included in the review

Study	Country	Study type	Study duration (year)	Sample size	Frequency	Gender Male	Average age	Evolutionary time in months	Monoarticular	Oligoarticular	Polyarticular	MSU	Uricemia(µmol/l)	Tophus	Diagnosis criteria
Diomandé M et al., (2022) [7]	Ivory cost	Retrospective	10	106	2,55	90	57,07	72,37	24	28	54	NP	512,52	37	Rome, New York, ACR
Kemta Lekpa F et al., (2016) [10]	Cameroon	Transversal	10	511	5,02	415	55,93	NP	172	195	137	NP	490,16	110	ACR
Gongnet P et al., (2022) [11]	Burkina Faso	Retrospective et Transversal	14	425	1,8	NP	56,3	40,92	NP	NP	NP	NP	NP	60	NP
Diallo M.L. et al., (2023) [12]	Togo	Retrospective	10	88	1,79	78	53,65	NP	10	54	24	5	488,61	17	ACR/EULAR
Touré. M.I et al., (2022) [13]	Mali	Prospective	1	69	11,87	57	52,7	NP	24	28	17	NP	547,26	5	ACR
Diallo S et al., (2017) [14]	Senegal	Retrospective	15	116	NP	102	55,7	NP	23	30	63	NP	NP	NP	NP
Fianyo E et al., (2023) [15]	Togo	Transversal	2	43	NP	41	58,83	64,63	NP	NP	NP	NP	534,35	NP	NP
Kemta Lekpa F et al., (2023) [16]	Cameroon	Transversal	1	111	5,69	107	51	NP	NP	NP	NP	NP	NP	NP	ACR/ EULAR
Lutalo S.K (1985) [17]	Zimbabwe	Retrospective	10	15	0,74	13	41,5	NP	NP	NP	NP	NP	642,44	4	NP
Sylla C (2010) [18]	Mali	Transversal	1,17	100	8,75	55	57,32	NP	33	25	42	NP	525,64	NP	NP
Malemba J. J and al., (2008) [19]	DRC	Retrospective	14	221	9,3	212	49,7	NP	81	86	54	NP	NP	17	Ryckewaert’s
Zabsonre W.J.S et al., (2017) [20]	Burkina Faso	Retrospective	8	158	2	153	52,75	86,33	95	49	9	33	550,91	33	EULAR
Singwé-N.M aet al., (2009) [21]	Cameroon	Retrospective	3	139	NP	131	55,71	5	29	43	67	120	452,09	47	ACR
Barry A et al., (2022) [22]	Senegal	Retrospective	14	106	NP	92	55,72	134,04	23	30	53	17	NP	39	ACR
Bilkish C and al., (1994) [23]	South Africa	Retrospective	5	107	NP	93	50,5	40,8	40	30	37	62	NP	39	ACR
Mijiyawa M, (1995) [24]	Togo	Retrospective	4	106	3	105	45,1	98,4	63	27	16	18	568.7	20	ACR
Mody G.M and al., (1984) [25]	South Africa	Retrospective	5	19	0,00047	15	51	37,2	5	0	14	8	NP	9	ACR
Djaha K.J-M et al., (2020) [26]	Ivory Coast	Retrospective	7	42	1,6	37	57	NP	7	15	20	NP	NP	21	ACR
Lamini N.E et al., (2019) [27]	Congo	Transversal	5	75	NP	60	60	88,92	18	18	39	NP	475,88	6	ACR

MSU: Detection of monosodium urate crystals

NP : Not precise DRC: Democratic Republic of Congo

### On the epidemiological front

**Fig. 2:**
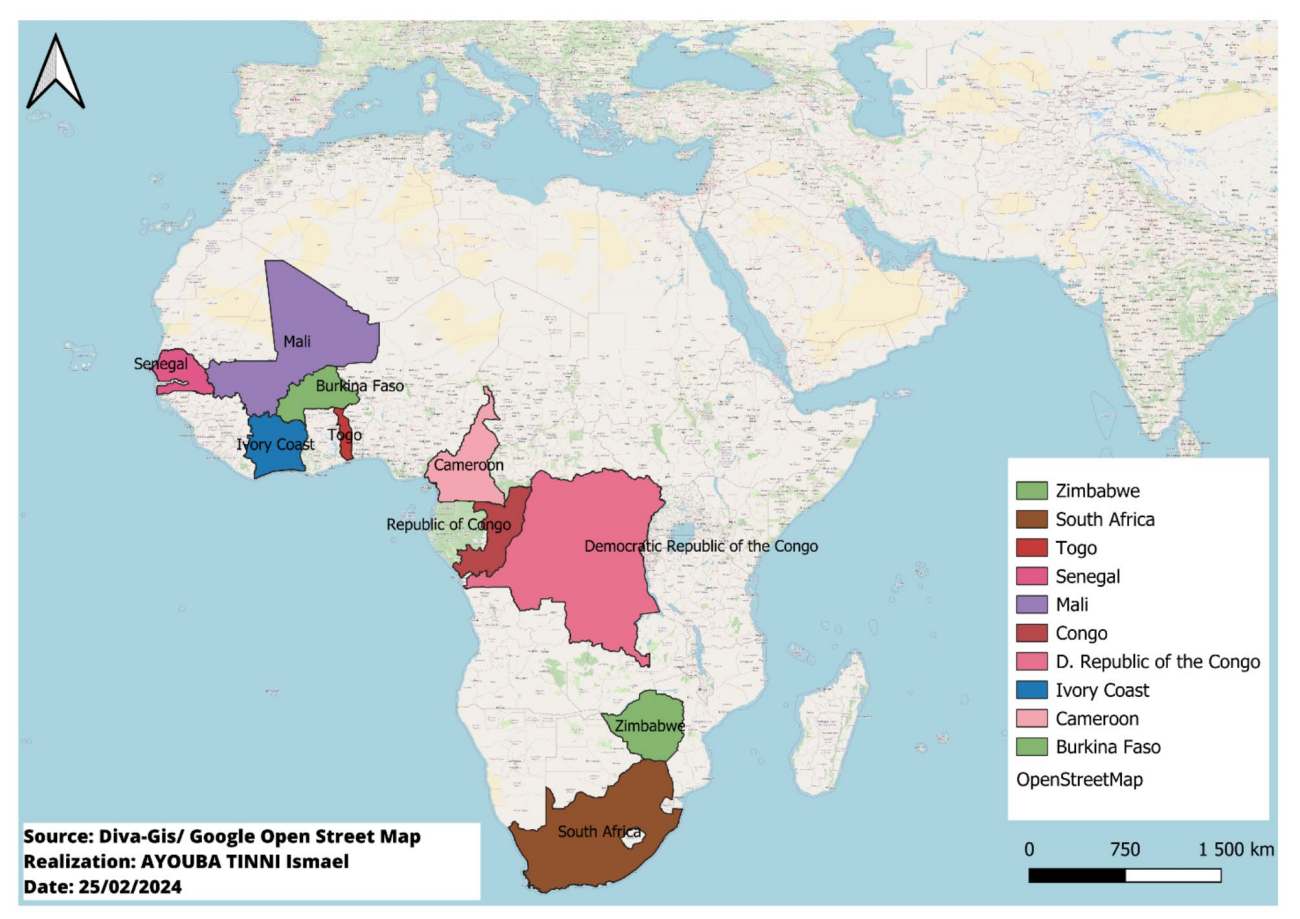
The geographical location of the countries in which the various articles have been published

The frequency of gout was reported in 13 studies, ranging from 0.00047 to 11.87%. In West Africa, the frequency of gout ranged from 1.6 to 11.87. In Central Africa, the frequency of gout ranged from 5.02 to 9.3%. In South Africa, the frequency of gout ranged from 0.00047 to 0.74. The highest frequencies were reported in Mali (11.87 and 8.75) and the Democratic Republic of Congo (9.3) [13, 18, 19]. Among the articles, 18 involving 2132 patients reported the sex of the patients, 1856 (87.05%) of whom were men. Nineteen articles reported the average age of patients, which ranged from 41.5 to 58.83 years, with an average age of 53.55 years. Ten studies reported the duration of the disease, ranging from 5 months to 134.04 months, with an average of 35.19 months. Figure 2 shows the geographical location of the countries in which the different types of articles were published.

### The diagnostic front

Five studies did not report how patients with gout were diagnosed [11, 12, 15, 17, 18]. Fourteen papers diagnosed gout using criteria, including nine studies with 1174 patients using the 1977 ACR criteria [28] in which participants either had proven gout by demonstration of monosodium urate crystals in joint fluid or tophus or met six of the 11 clinical criteria. The other five studies included in the review diagnosed patients with gout using the EULAR or ACR-EULAR criteria or a combination of the Rome, New York, and ACR criteria or Ryckewaert’s criteria [19, 28–32]. Fifteen articles reported the form of gout (monoarticular in 647 (33.16%) patients, oligoarticular in 658 (33.73%) patients and polyarticular in 646 (33.11%) patients in 1968). Gouty tophi were reported in 15 articles involving 464 patients. Of the studies reviewed, seven examined the presence of monosodium urate crystals in 317 (43.85%) patients out of a total of 723 gouty patients. Among the 317 patients, monosodium urate crystals were identified in 263 (82.97%). Eleven studies reported mean uricemia values ranging from 452.09 µmol/L to 642.44 µmol/L, with a mean of 510.63 µmol/L (Table 1). Standard radiography was reported in five articles for 395 patients.

## Discussion

The objective of this review was to synthesize studies that have addressed the epidemiological and diagnostic aspects of gout in sub-Saharan Africa. This synthesis will serve as an evidence base to facilitate the planning and conduct of future gout research. Of the 19 included studies, 12 were retrospective. The frequency of gout is variable in sub-Saharan Africa. The highest frequencies were reported in Mali (11.87 and 8.75) and the Democratic Republic of Congo (9.3) [13, 18, 19]. The 1977 ACR criteria [28] were used to diagnose gout in nine of the 19 studies included in the data analysis. Gout tophi were reported in 15 articles involving 464 patients. Of the studies included in the data analysis, seven examined the presence of monosodium urate crystals in 317 (43.8%) patients out of a total of 723 gouty patients. Among the 317 patients, monosodium urate crystals were identified in 263 (82.97%).

Gouts in sub-Saharan Africa have the same demographic and semiological characteristics as those in the West. There is a clear male predominance of over 80%. Onset is usually between 41.5 and 58.83 years of age, with a mean age of 53.55 years. It is relatively common in rheumatology in sub-Saharan Africa, as shown by its frequency, which ranged from 0.00047 to 11.87%, and sample sizes, which ranged from 15 to 511 patients. This shows that sub-Saharan Africa is not spared from gout, which has long been considered rare, if not exceptional, in black African populations. This assertion is all the more credible given that the condition is often considered to be the preservation of the rich and overfed [5, 6, 20, 33]. However, the increase in gout cases may be due to increased life expectancy, the use of certain medications (diuretics, low-dose aspirin, pyrazinamide) and comorbidities (cardiovascular disease, hypertension, metabolic syndrome), as well as changes in diet [6, 33–37]. Several studies have shown that diet is a risk factor for gout [6, 37]. According to a follow-up study of American healthcare professionals, an increase in the daily intake of meat or seafood products was associated with an increase in the incidence of gout, while dairy products had a protective effect by promoting the excretion of uric acid [6, 38]. More recently, it has been shown that consumption of more than two regular or fructose-sweetened soft drinks or fruit juices rich in fructose increases the risk of gout; that moderate consumption of vegetables rich in purines or proteins does not increase this risk; and that consumption of coffee and vitamin C reduces this risk [6, 37, 38].

The discrepancy between the sample sizes of the different studies is probably due to poor health care coverage and numerous recruitment biases: lack of uniformity in the diagnostic criteria used by the different authors, patients not systematically consulting care centers, and lack of population surveys [5]. Moreover, uricemia may be normal during gouty attacks, making the diagnosis even more difficult, not to mention the difficulty of detecting monosodium urate crystals, which were found in 263 (82,97%) of the 723 patients in our study. These series were compiled on the basis of the ACR 1077, New York, Rome, and other criteria. A recent study attempted to validate these clinical criteria by relating them to the presence of crystals [31, 39]. None of the three groups of criteria (ACR 1977, New York, and Rome) had a sensitivity greater than 70% or a specificity greater than 78.8%. This work has only shown that these criteria allow the exclusion of gout, but they do not replace the presence of monosodium urate crystals in a fluid and cannot therefore be used as diagnostic criteria [31, 39–41]. In addition, the mean duration of the disease ranged from five months to 134.04 months; oligoarticular and polyarticular forms accounted for 2/3 of patients, and gouty tophi were reported in 464 patients, suggesting a delay in diagnosis, especially as the time from asymptomatic hyperuricemia to chronic tophaceous gout is quite variable (from 3 to 42 years, with an average of 11 years) [6, 42]. In addition, the mean uricemia levels ranged from 452.09 µmol/L to 642.44 µmol/L. Uric acid levels play a role in the risk of developing gout, although approximately 10% of hyperuricaemia patients will develop gout, suggesting that other factors play a role in the development of this disease [6, 43]. In the paraclinical setting, standard radiography was reported in 5 articles with 395 patients. This low rate of radiography may be explained by the fact that radiographic signs appear late and do not allow early diagnosis [44]. This analysis of the literature has enabled us to note the almost non-existent use of ultrasound for the diagnosis of gout in sub-Saharan Africa, even though it contributes to the diagnosis of gout by highlighting specific signs testifying to deposits of microcrystals in joints and periarticular structures (double-contour appearance, “snowstorm” appearance, intra- or periarticular tophus, and erosion) [44]. In addition, double-contour appearance is among the gout classification criteria developed by the American College of Rheumatology/European League Against Rheumatism (ACR/EULAR) in 2015 [44,45]. This lack of use of ultrasound for the diagnosis of gout in sub-Saharan Africa can be explained by the fact that this technique is recent, and in addition, the totality of diagnostic criteria used by the various studies analysed did not classify ultrasound signs as diagnostic criteria. It should also be noted that ultrasound is an operator-dependent examination. Ultrasound equipment is not available in most rheumatology departments in sub-Saharan Africa. There is also a lack of personnel who can reliably perform ultrasound scans for signs of gout.

This study has a number of limitations that should be acknowledged. First, the literature search was limited to online journals. Consequently, only full-text articles were included, which may have excluded potentially relevant information. In addition, the analysis was limited to articles published in English or French, which excluded studies published in other languages.

## Conclusions

This review shows that all studies of gout in sub-Saharan Africa have been hospital-based, with identical clinical presentations in different countries, and highlights the need for future population-based or multicenter and multicountry studies based on a rigorous methodology to determine the exact prevalence, risk factors, and severity of gout in sub-Saharan Africa. This study will provide a database for the sub-Saharan region and facilitate future therapeutic trials to improve the management of gout patients in our context.

### Electronic supplementary material

Below is the link to the electronic supplementary material.

Supplementary Material 1

## Data Availability

Data supporting the results of this study are available from the corresponding author on reasonable request.
