# Supplementary material for: Epidemiology and diagnosis of gout in sub-saharan Africa: a scoping review
Source: BMC Rheumatol. 2024 May 23;8:21. doi: 10.1186/s41927-024-00391-w (PMC11112960; doi:10.1186/s41927-024-00391-w)
Supplement: Supplementary file 1 — Supplementary Material 1 [file 41927_2024_391_MOESM1_ESM.docx]

**Additional file 1**: Information on Sub-Saharan Africa region

**Sub-Saharan Africa**, **Subsahara**, or **Non-Mediterranean Africa** is the area and regions of the continent of [Africa](https://en.wikipedia.org/wiki/Africa) that lie south of the [Sahara](https://en.wikipedia.org/wiki/Sahara). These include [Central Africa](https://en.wikipedia.org/wiki/Central_Africa), [East Africa](https://en.wikipedia.org/wiki/East_Africa), [Southern Africa](https://en.wikipedia.org/wiki/Southern_Africa), and [West Africa](https://en.wikipedia.org/wiki/West_Africa).

**Central Africa** is a [subregion](https://en.wikipedia.org/wiki/Subregion) of the [African continent](https://en.wikipedia.org/wiki/African_continent) comprising various countries according to different definitions. **Middle Africa** is an analogous term used by the United Nations in its [geoscheme for Africa](https://en.wikipedia.org/wiki/United_Nations_geoscheme_for_Africa) and consists of the following countries : [Angola](https://en.wikipedia.org/wiki/Angola), [Burundi](https://en.wikipedia.org/wiki/Burundi), [Cameroon](https://en.wikipedia.org/wiki/Cameroon), [Central African Republic](https://en.wikipedia.org/wiki/Central_African_Republic), [Chad](https://en.wikipedia.org/wiki/Chad), [Democratic Republic of the Congo](https://en.wikipedia.org/wiki/Democratic_Republic_of_the_Congo), [Republic of the Congo](https://en.wikipedia.org/wiki/Republic_of_the_Congo), [Equatorial Guinea](https://en.wikipedia.org/wiki/Equatorial_Guinea), [Gabon](https://en.wikipedia.org/wiki/Gabon), [Rwanda](https://en.wikipedia.org/wiki/Rwanda), and [São Tomé and Príncipe](https://en.wikipedia.org/wiki/S%C3%A3o_Tom%C3%A9_and_Pr%C3%ADncipe).

**East Africa** refers to the area comprising [Kenya](https://en.wikipedia.org/wiki/Kenya), [Tanzania](https://en.wikipedia.org/wiki/Tanzania), and [Uganda](https://en.wikipedia.org/wiki/Uganda), largely due to their shared history under the [Omani Empire](https://en.wikipedia.org/wiki/Omani_Empire) and as parts of the [British East Africa Protectorate](https://en.wikipedia.org/wiki/British_East_Africa_Protectorate) and [German East Africa](https://en.wikipedia.org/wiki/German_East_Africa). In broader linguistic and geographic interpretations, the term encompasses additional countries such as [Djibouti](https://en.wikipedia.org/wiki/Djibouti), [Eritrea](https://en.wikipedia.org/wiki/Eritrea), [Ethiopia](https://en.wikipedia.org/wiki/Ethiopia), and [Somalia](https://en.wikipedia.org/wiki/Somalia).

**Southern Africa** includes both [subtropical](https://en.wikipedia.org/wiki/Subtropics) and [temperate](https://en.wikipedia.org/wiki/Temperate_climate) climates, with the [Tropic of Capricorn](https://en.wikipedia.org/wiki/Tropic_of_Capricorn) running through the middle of the region, dividing it into its subtropical and temperate halves. Countries commonly included in Southern Africa include [Angola](https://en.wikipedia.org/wiki/Angola), [Botswana](https://en.wikipedia.org/wiki/Botswana), the [Comoros](https://en.wikipedia.org/wiki/Comoros), [Eswatini](https://en.wikipedia.org/wiki/Eswatini), [Lesotho](https://en.wikipedia.org/wiki/Lesotho), [Madagascar](https://en.wikipedia.org/wiki/Madagascar), [Malawi](https://en.wikipedia.org/wiki/Malawi), [Mauritius](https://en.wikipedia.org/wiki/Mauritius), [Mozambique](https://en.wikipedia.org/wiki/Mozambique), [Namibia](https://en.wikipedia.org/wiki/Namibia), [South Africa](https://en.wikipedia.org/wiki/South_Africa), [Zambia](https://en.wikipedia.org/wiki/Zambia), and [Zimbabwe](https://en.wikipedia.org/wiki/Zimbabwe). In [cultural geography](https://en.wikipedia.org/wiki/Cultural_geography), the island country of [Madagascar](https://en.wikipedia.org/wiki/Madagascar) is often not included due to its distinct language and cultural heritage.

**West Africa** or **Western Africa** is the westernmost region of [Africa](https://en.wikipedia.org/wiki/Africa). The [United Nations defines](https://en.wikipedia.org/wiki/United_Nations_geoscheme_for_Africa#Western_Africa) Western Africa as the 16 countries of [Benin](https://en.wikipedia.org/wiki/Benin), [Burkina Faso](https://en.wikipedia.org/wiki/Burkina_Faso), [Cape Verde](https://en.wikipedia.org/wiki/Cape_Verde), [The Gambia](https://en.wikipedia.org/wiki/The_Gambia), [Ghana](https://en.wikipedia.org/wiki/Ghana), [Guinea](https://en.wikipedia.org/wiki/Guinea), [Guinea-Bissau](https://en.wikipedia.org/wiki/Guinea-Bissau), [Ivory Coast](https://en.wikipedia.org/wiki/Ivory_Coast), [Liberia](https://en.wikipedia.org/wiki/Liberia), [Mali](https://en.wikipedia.org/wiki/Mali), [Mauritania](https://en.wikipedia.org/wiki/Mauritania), [Niger](https://en.wikipedia.org/wiki/Niger), [Nigeria](https://en.wikipedia.org/wiki/Nigeria), [Senegal](https://en.wikipedia.org/wiki/Senegal), [Sierra Leone](https://en.wikipedia.org/wiki/Sierra_Leone), and [Togo](https://en.wikipedia.org/wiki/Togo), as well as [Saint Helena, Ascension and Tristan da Cunha](https://en.wikipedia.org/wiki/Saint_Helena,_Ascension_and_Tristan_da_Cunha) ([United Kingdom Overseas Territory](https://en.wikipedia.org/wiki/United_Kingdom_Overseas_Territories)).

References

1. ["World Population Prospects 2022"](https://population.un.org/wpp/). [United Nations Department of Economic and Social Affairs](https://en.wikipedia.org/wiki/United_Nations_Department_of_Economic_and_Social_Affairs), Population Division. Retrieved 17 July 2022.

2. [Jump up to:**^a^**](https://en.wikipedia.org/wiki/West_Africa#cite_ref-UN_WPP_2022_2-0) [**^b^**](https://en.wikipedia.org/wiki/West_Africa#cite_ref-UN_WPP_2022_2-1) [**^c^**](https://en.wikipedia.org/wiki/West_Africa#cite_ref-UN_WPP_2022_2-2) ["World Population Prospects 2022: Demographic indicators by region, subregion and country, annually for 1950-2100"](https://population.un.org/wpp/Download/Files/1_Indicators%20(Standard)/EXCEL_FILES/1_General/WPP2022_GEN_F01_DEMOGRAPHIC_INDICATORS_COMPACT_REV1.xlsx) (XSLX) ("Total Population, as of 1 July (thousands)"). [United Nations Department of Economic and Social Affairs](https://en.wikipedia.org/wiki/United_Nations_Department_of_Economic_and_Social_Affairs), Population Division. Retrieved 17 July 2022.
